# Surveying the Metabolic and Dysfunctional Profiles of T Cells and NK Cells in Myalgic Encephalomyelitis/Chronic Fatigue Syndrome

**DOI:** 10.3390/ijms241511937

**Published:** 2023-07-26

**Authors:** Jessica Maya

**Affiliations:** Department of Molecular Biology and Genetics, Cornell University, Ithaca, NY 14850, USA; jm2629@cornell.edu

**Keywords:** myalgic encephalomyelitis, chronic fatigue syndrome, immune cell dysfunction, immunometabolism, T cells, NK cells, T cell exhaustion

## Abstract

Millions globally suffer from myalgic encephalomyelitis/chronic fatigue syndrome (ME/CFS). The inflammatory symptoms, illness onset, recorded outbreak events, and physiological variations provide strong indications that ME/CFS, at least sometimes, has an infectious origin, possibly resulting in a chronic unidentified viral infection. Meanwhile, studies exposing generalized metabolic disruptions in ME/CFS have stimulated interest in isolated immune cells with an altered metabolic state. As the metabolism dictates the cellular function, dissecting the biomechanics of dysfunctional immune cells in ME/CFS can uncover states such as exhaustion, senescence, or anergy, providing insights into the consequences of these phenotypes in this disease. Despite the similarities that are seen metabolically between ME/CFS and other chronic viral infections that result in an exhausted immune cell state, immune cell exhaustion has not yet been verified in ME/CFS. This review explores the evidence for immunometabolic dysfunction in ME/CFS T cell and natural killer (NK) cell populations, comparing ME/CFS metabolic and functional features to dysfunctional immune cell states, and positing whether anergy, exhaustion, or senescence could be occurring in distinct immune cell populations in ME/CFS, which is consistent with the hypothesis that ME/CFS is a chronic viral disease. This comprehensive review of the ME/CFS immunometabolic literature identifies CD8+ T cell exhaustion as a probable contender, underscores the need for further investigation into the dysfunctional state of CD4+ T cells and NK cells, and explores the functional implications of molecular findings in these immune-cell types. Comprehending the cause and impact of ME/CFS immune cell dysfunction is critical to understanding the physiological mechanisms of ME/CFS, and developing effective treatments to alleviate the burden of this disabling condition.

## 1. Introduction

### 1.1. An Overview of ME/CFS

The disabling condition known as ME/CFS afflicts roughly 65 million people worldwide [1]. Currently, the causal agents remain controversial, and there is no approved diagnostic test, nor any FDA-approved treatment for individuals with ME/CFS [2]. The onset of this illness, averaging at an age of 33, can occur over time or suddenly, but many individuals remain undiagnosed for years [2,3]. Even though most scientific investigations and interventional studies on ME/CFS consist of participants who are primarily Caucasian, community health studies suggest that the illness may be more widespread, and have a more severe symptom presentation, in minority populations, and in individuals from lower socioeconomic classes [4]. The level of severity in ME/CFS can fluctuate greatly, with some individuals experiencing such debilitating symptoms that they are rendered immobile, confined to their homes or beds, and forced to endure the burden of this condition without respite for months, years, or decades [2].

ME/CFS disease manifestation encompasses a range of disabling symptoms that dramatically reduce an individual’s quality of life. The most prominent symptoms include severe fatigue, sleep disturbances, gastrointestinal symptoms, post-exertional malaise, orthostatic intolerance, and cognitive impairments, such as attention deficits, a poor working memory, and complex information-processing impairments [2]. In addition, people with ME/CFS commonly report immune-related or inflammatory symptoms, such as headaches, muscular pain, tender lymph nodes, and fever [5]. This cluster of symptoms has been compared to a perpetual flu-like state [6].

Despite this illness presentation, and other significant reports implying the involvement of an infectious event, the triggering agent remains a topic of debate in the scientific community [7,8,9,10]. Several viruses have been implicated in ME/CFS, including, but not limited to, the Epstein–Barr virus (EBV), human herpesvirus 6 (HHV-6), and enteroviruses A and B (EV-A and EV-B) [7,8,10,11,12,13,14,15,16]. While the mechanisms by which one or more of these viruses may contribute to the development of ME/CFS are not fully understood, other studies indicate that chronic viral infections can cause long-term changes in the immune system [17] that are similar to those observed in ME/CFS. 

### 1.2. Implicated Viral Infections in ME/CFS

**Figure 1:**
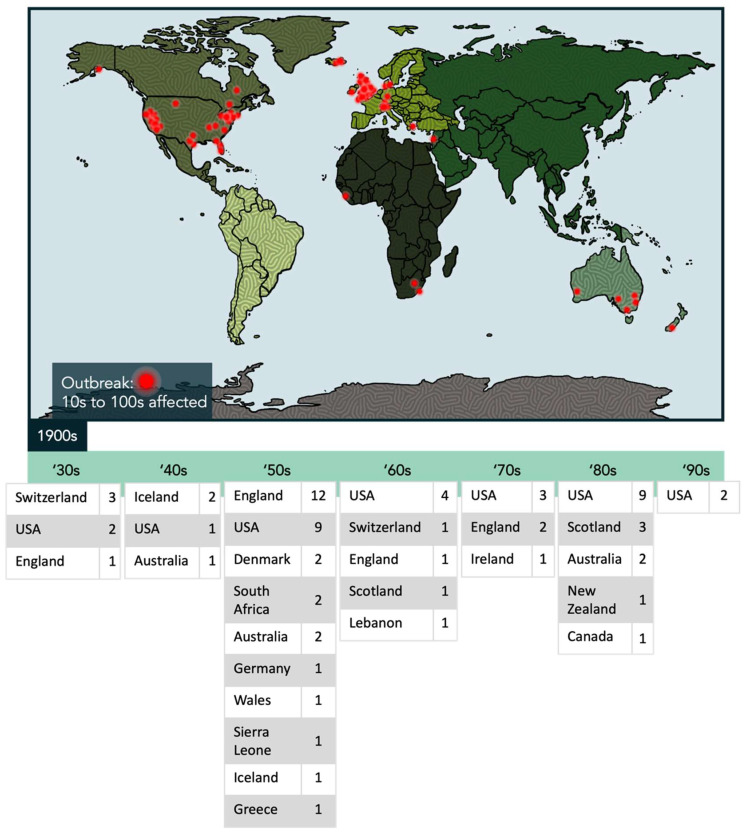
Recorded outbreaks of ME/CFS during the 1900s. Documented ME/CFS outbreaks worldwide, spanning the 1930s to the 1990s, are indicated by a red dot on the map, which represents between tens and hundreds of individuals afflicted with ME/CFS during each outbreak. The tables below the map display the number of outbreaks by country, and within each decade of this timeline [18,19,20,21,22].

In the past century, ~74 documented clustered outbreaks of ME/CFS have been observed worldwide, their identification likely limited to certain regions in part due to poor or unavailable records at the time (Figure 1) [18,19,20,21,22]. These outbreaks affected between tens and hundreds of individuals at a time, and while some people did recover, others remained ill for years [18,20]. During this time, and in subsequent decades, the disease carried many different names, either based on the outbreak location, or the disease manifestation [19]. The descriptor “myalgic encephalomyelitis” was first introduced during a 1950s outbreak at the Royal Free Hospital in London, as myalgic denotes muscle pain, and encephalomyelitis specifies the inflammation of the brain and spinal cord [23]. Despite two major outbreaks in the US in the 1980s, leading to the Center for Disease Control (CDC) coining the term “chronic fatigue syndrome”, the name most commonly used by the affected community is the umbrella term “ME/CFS” [18]. 

ME/CFS is often preceded by flu-like symptoms, and many patient accounts describe a viral-like illness or an identified infection around the time of onset [3]. Although the root cause of this condition is still uncertain, numerous studies have linked ME/CFS to viruses [7,8,10,11,12,13,14,15,16]. However, identifying the specific virus responsible for the development of ME/CFS has proven to be a difficult challenge. One of the difficulties in identifying the inciting virus in ME/CFS is the lack of a singular, straightforward diagnostic test. ME/CFS diagnosis relies on multiple criteria, often involving the exclusion of other diseases or the presence of specific symptoms over an extended period [2,18,24]. Consequently, the time between disease onset and diagnosis averages seven years [2]. Moreover, unlike acute infections, where viral particles can be readily detected in blood or other bodily fluids, chronic viral infections can be much more difficult to diagnose. This is because the viral load in the body may be much lower in chronic infections, and the virus may not be detectable in routine laboratory tests [25,26]. For example, if the virus is a variant, the sequence may not be properly amplified for PCR detection, preventing identification [25]. Furthermore, there is no consensus on what constitutes a “positive” test for many of the viruses that have been implicated in ME/CFS, which can lead to confusion and inconsistencies in the interpretation of test results.

Among the viral candidates associated with ME/CFS, the Epstein–Barr virus (EBV), human herpesvirus 6 (HHV-6), and enteroviruses emerge as the most frequently implicated [7,27,28]. For example, some individuals with ME/CFS display distinct EBV antibody levels, defective EBV-specific immune cell responses, and higher levels of anti-EBV-dUTPase antibodies and active EBV infection compared to healthy controls [2,29,30,31,32], making some scientists hypothesize that the EBV infection is evading the immune system in ME/CFS [27]. A higher prevalence of active HHV-6 has also been observed in ME/CFS patients [33,34,35], along with higher antibody levels of HHV-6 in some ME/CFS cohorts [16,36,37], and an abundance of active HHV-6 transcripts in ME/CFS brain and spinal cord samples [38]. Although there is evidence that links HHV-6 and ME/CFS, it is possible that this virus plays only a contributing role, by increasing an individual’s susceptibility to ME/CFS, or by sustaining the condition once it is established. Enteroviruses, such as EV-A and EV-B, are associated with ME/CFS, sharing symptomatology and seasonal incidence patterns [8,20]. ME/CFS biopsies have revealed an increased enterovirus presence in the muscles and stomach, along with higher serum antibodies, when compared to healthy controls [10,15,39,40,41]. Interestingly, a study by John Chia’s group found that stomach biopsies from individuals with ME/CFS could infect immunodeficient mice [42]. Overall, EBV, HHV-6, and enteroviruses demonstrate potential roles in ME/CFS pathogenesis, immune cell dysfunction, and symptom manifestation, requiring further research to unravel the viral mechanisms underlying this complex condition.

Further confirmation of viral involvement in this disease is evidenced by the response of some patients to antiviral therapies and other immunomodulatory treatments. One six-month treatment study with valacyclovir, an antiviral drug, resulted in an increased physical functional capacity and improved cardiac function in patients within an EBV+ ME/CFS classification [13]. Similar positive responses were observed in a six-month trial of another antiviral, valganciclovir, in a separate ME/CFS cohort [43]. A third antiviral study demonstrated that Isoprinosine, which works to amplify the natural immune response, was successful in increasing natural killer (NK) cell activity and IL-12 production in an ME/CFS cohort, subsequently improving clinical health measurements in the majority of the patient participants [44]. In both adolescent and adult patients, several trials have shown that intravenous immunoglobulin holds promise as a treatment option, potentially due to its ability to provide immunomodulatory effects that regulate the immune response, and prevent excessive inflammation [45,46,47,48]. 

The ongoing COVID-19 pandemic has highlighted the similarities between ME/CFS and post-acute SARS-CoV-2 syndrome, which present with overlapping symptoms, providing further evidence of a viral onset in ME/CFS [49]. However, conflating the two illnesses can lead to confusion and potential harm in the ME/CFS research community. A recent genome-wide association study suggested that there was no significant genetic relationship between COVID-19 severity, hospitalization, and susceptibility to ME/CFS, but rather that these conditions share similar symptoms related to immune system dysregulation, and a heightened inflammatory response [50,51]. Moreover, hypercoagulability, microclot loads, and hyperactivated platelets are extensive in post-acute SARS-CoV-2 syndrome, but are observed less frequently in pre-2020 ME/CFS cases [52,53]. It is crucial to recognize and address the distinction between these two illnesses, particularly when developing treatment strategies. Although anti-SARS-CoV-2 vaccines have been reported to alleviate symptoms in some long-term COVID-19 patients, such a treatment will not be effective for individuals with ME/CFS, as the viral agent involved is unquestionably different [54]. 

## 2. Metabolic Dynamics in T and NK Cell Populations

At the onset of viral exposure, an infection can either be cleared or become chronic, and various factors, including dysfunctional immune cell states that alter the balance of T cell sub-populations, and cause a pro-inflammatory shift, can influence the transition to, and maintenance of, a chronic infection [55]. Certain viruses have developed unique strategies that enable them to persist for the lifetime of the host: in some cases, evading the immune system to continually replicate and stimulate the immune response, while in other examples, entering latency periods with sporadic reactivation events [55]. These chronic viral infections can have long-term effects on the immune system [17]. Importantly, the persistent stimulation of the immune system, and continuous exposure to viral products can make an individual more vulnerable to secondary infections, cancers, and long-lasting inflammation [17]. Such prolonged effects on the immune system may sustain the symptoms of ME/CFS. 

Exploring the bioenergetic mechanisms responsible for immune dysfunction can offer insight into the onset of disease, and facilitate the development of innovative strategies to manage infections or reverse impaired immune cell states. I will present an overview of immunometabolism and its critical role in immune function, review the current literature on immunometabolic differences in ME/CFS, and discuss the altered metabolic programming that drives dysregulated immune cell phenotypes during chronic antigen stimulation. Lastly, I will discuss the possible immune cell dysfunction observed in ME/CFS, based on the metabolic and functional literature on dysregulated T and NK cell states.

### 2.1. Immunometabolism in Classic T and NK Cells

**Figure 2:**
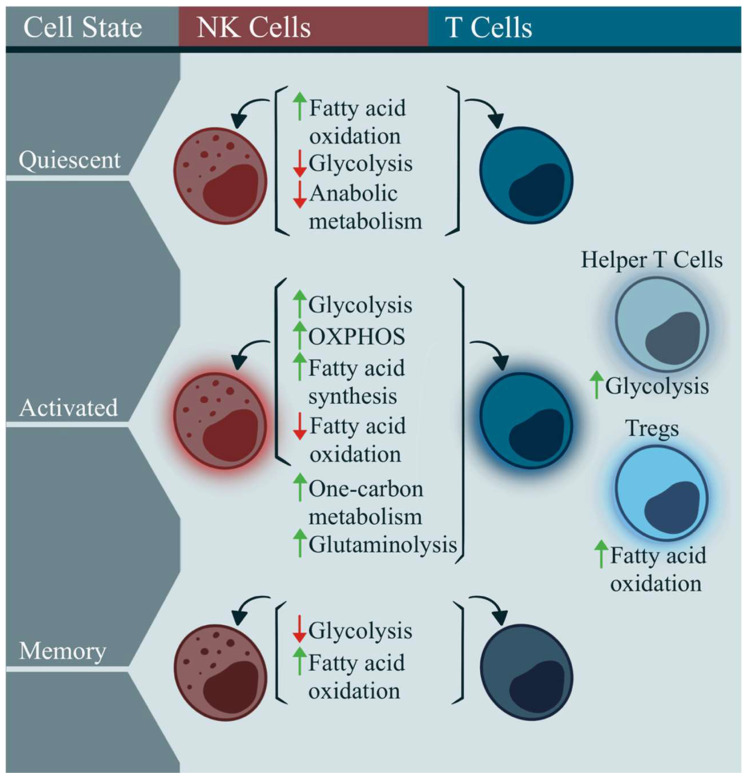
Metabolism in the NK cells and T cells during the quiescence, activation, and memory states. Resting lymphocytes predominantly rely on fatty acid oxidation to maintain low energy requirements [58,59,60,61,62]. Upon activation, lymphocytes increase the substrate import, and synthesize various metabolites to support biomass for proliferation and the effector function, increasing glycolysis, OXPHOS, and fatty acid synthesis, while reducing fatty acid oxidation [58,59,60,61,62,63,64,65,66,67]. However, metabolic shifts can differ for different immune cell populations. For example, glutaminolysis and one-carbon metabolism may not be required for NK cell activation, while Tregs have been found to rely on fatty acid oxidation, rather than glycolysis, to balance the immune response [68,69,70,71]. After the infection clearance, memory cells upregulate fatty acid oxidation, to sustain themselves for potential reactivation [59,72].

T cells are an essential component of the adaptive immune response, and can be divided into two main types: CD4+ T cells, also called “helper” cells, and CD8+ T cells, also known as “cytotoxic” cells [56]. Another type of cytotoxic immune cell found in the innate immune response is an NK cell; these are well-known for their ability to destroy virally infected cells, and to detect and control early cancer cells [57]. In healthy individuals, the immune cell metabolism is a precisely regulated system that adapts to promote specific functions, and meet energy demands (Figure 2). Circulating naïve T cells and NK cells preferentially generate energy through catabolic metabolism, relying primarily on fatty acid oxidation (FAO), with minimal glucose metabolism, to fuel the subsequent oxidative phosphorylation (OXPHOS) (Figure 2) [58,59,60,61,62]. 

Once activated, immune cells upregulate OXPHOS and glycolysis, to produce large amounts of energy for effector molecule and biomass production (Figure 2) [59,60]. Specifically, when T cell receptors (TCR) on a T cell bind to a cognate antigen on an antigen-presenting cell, signals through the c-Myc and PI3K/AKT/mTOR pathways increase glycolysis, while also increasing fatty acid synthesis, and lowering FAO [62,73,74]. This is achieved by increasing the protein degradation and amino acid uptake [75,76]. The one-carbon metabolism and glutaminolysis are also increased, to provide intermediates that ultimately enhance proliferation and survival (Figure 2) [77,78]. 

Similarly, NK cells will utilize PI3K/AKT/mTOR signaling to remodel their metabolism once IL-2, IL-12, IL-15, or IL-18 activates the cell, subsequently increasing fatty acid synthesis, glycolysis, and OXPHOS, while decreasing FAO (Figure 2) [63,64,65,66,67]. While excess one-carbon metabolites in plasma have been found to decrease NK cell cytotoxicity, it is unclear whether the metabolism of these fatty acids, specifically within NK cells, can promote the effector functions [68]. The mitochondrial mass is increased following NK cell activation [79]. Glutamine has not been found to be an important factor in NK cell activation [69]. 

Different subsets of T cells can rely on specific metabolic pathways based on their effector or suppressive functional state—CD4+ helper cells rely on glycolysis for their effector function, whereas regulatory T cells (Tregs) depend on fatty acids to fuel anti-inflammatory responses (Figure 2) [70,71]. Post-infection, memory cells among both T and NK cell populations downregulate glycolysis, and rely on FAO for long-term maintenance (Figure 2) [59,72]. 

### 2.2. Immunometabolism in ME/CFS

Several studies have investigated the immune metabolism in ME/CFS through the analysis of the peripheral blood mononuclear cells (PBMCs), which include T cells, B cells, NK cells, dendritic cells, and monocytes. Metabolic assays of PBMCs in ME/CFS patients have demonstrated several alterations in the energy pathways and mitochondrial function, including reduced glycolysis, decreased mitochondrial respiration, lower coupling efficiency, and changes in the pyruvate dehydrogenase and CoA metabolism, all of which suggest insufficient ATP production, and the dysregulation of fatty acid oxidation [80,81,82,83]. In fact, a decreased ATP production was seen in two studies of ME/CFS PBMCs, with one of these studies also reporting a reduction in the coenzyme Q10 in ME/CFS [80,84]. Nevertheless, there are no significant differences in the PBMC mitochondrial complex activity in individuals with ME/CFS [82,85,86,87]. While one study reported a decrease in the mitochondrial mass in ME/CFS PBMCs, another found no difference, compared to healthy controls [84,86]. Interestingly, a separate study on ME/CFS PBMCs showed a significant increase in non-mitochondrial ATP production, and observed an increased mitochondrial cristae condensation, but no differences in the mitochondrial morphology or membrane potential [85]. Further, proteomic analyses of PBMCs and immortalized lymphoblasts in ME/CFS patients have described an increased expression of the enzymes involved in ketone body metabolism, a greater expression of acyl-CoA dehydrogenases, and upregulated levels of the enzymes mediating fatty acid oxidation [88,89,90]. Despite this evidence pointing toward an altered metabolism in ME/CFS PBMCs compared to healthy controls, the precise mechanism driving this dysfunction remains unknown, thus emphasizing the importance of exploring the metabolism of isolated immune cells, given the heterogeneity of PBMCs.

We have previously investigated the isolated T cell metabolism in ME/CFS, with a focus on glycolysis, oxidative phosphorylation, and fatty acid oxidation. CD4+ and CD8+ T cells exhibited reduced basal glycolysis at rest, seen again in CD8+ T cells after stimulatory factors were added [91]. Additionally, ME/CFS CD8+ T cells demonstrated a decreased proton leak, reduced ATP production, and lower mitochondrial membrane potential, the last of which was observed both at rest, and following activation compared to healthy control cells [91]. Moreover, both the CD4+ and CD8+ T cells exhibited an elevated FAO in ME/CFS, compared to the healthy control cells [92].

Otherwise, the metabolism in ME/CFS T cells remains largely unexplored. Inferences can be made, however, from T cell functional deficits that have been recorded in this illness. For example, ME/CFS CD4+ T cells with an increased expression of the inhibitory receptor programmed cell death protein 1 (PD-1) have been reported; this protein can cause cells to enter a hypometabolic state, while increasing fatty acid oxidation [93]. The reduced cytotoxicity observed in ME/CFS CD8+ T cells [94,95] also implies a similarly altered metabolic dynamic. Furthermore, a flow cytometric analysis of T cell populations revealed an increased CD4+ to CD8+ T cell ratio, and an increased CD8+ effector memory T cell frequency in ME/CFS patients under 50 years of age [96]. CCR6+ Th17 cells in ME/CFS produced less IL-17 than controls, while their frequency was higher [96]. Meanwhile, the mucosal-associated invariant T (MAIT) cells secreted lower IFNγ, GranzymeA, and IL-17 after activation in ME/CFS samples, indicating the chronic stimulation of these T cell populations [96]. The persistent stimulation and decreased effector function in these cell populations imply a hypometabolic state in ME/CFS T cells [97]. Furthermore, the extracellular environment of these cells likely influences the cellular metabolism. Plasma metabolomic studies consistently report altered lipid abundances and, specifically, increased carnitine compounds, further corroborating the fatty acid oxidation irregularities in ME/CFS [98,99,100,101,102,103]. 

In ME/CFS NK cells, a pilot study described a reduced glycolytic reserve compared to healthy control cells, implying that ME/CFS NK cells may be unable to respond as effectively to energy-demanding situations, such as during an infection or immune challenge, compared with their healthy counterparts [104]. Additionally, a study on NK cell metabolism suggested that there was no difference in glycolysis, GLUT1, mitochondrial respiration, mitochondrial membrane potential, or mitochondrial mass between ME/CFS and healthy control NK cells, at rest or following stimulation [105]. Furthermore, I recently reported that the FAO was increased in ME/CFS NK cells, but extracellular fatty acids were limited in their entry to these cells, compared to the healthy control samples [92].

The understanding of NK cell metabolism in ME/CFS is still limited, but the dissection of cellular function can still provide useful insights. A common and well-established finding in ME/CFS is that the NK cell cytotoxicity is decreased [94,95,106,107,108,109]. As the cytotoxic function in NK cells relies on OXPHOS, the reduced effect that has been observed could be due to a lower rate of OXPHOS in ME/CFS NK cells [65]. Additionally, the excess lipid accumulation in the extracellular environment, established by many ME/CFS metabolomic studies mentioned previously, is known to clog the NK cell cytotoxic machinery, and suppress mTOR activity [79,110,111]. Specifically, studies have reported lower levels of granzyme B production in ME/CFS, which is known to require mTORC1 for proper synthesis [63]. These findings indicate that metabolic reprogramming in the NK cells may underlie the observed cytotoxic dysfunction in ME/CFS.

Although other immune cell types have been studied in ME/CFS, such as B cells, neutrophils, dendritic cells, and monocytes, their metabolic dynamics have not been thoroughly investigated, and are beyond the scope of this review.

### 2.3. Immunometabolism in Dysfunctional Immune Cell States

In dysfunctional states, the activated immune cells responsible for critical effector functions can have metabolic underpinnings that impede the proper immune cell proliferation and function [112]. These metabolic changes, whether programmed or pathological, can interfere with the cell’s ability to meet the elevated bioenergetic demands and increased fuel utilization required for an efficient and effective immune response [112]. The terms ignorance, anergy, tolerance, exhaustion, and senescence have been inconsistently used in the literature to describe various states of lymphocyte dysfunction, mainly in the context of T cells, leading to confusion within the field (Table 1) [113]. However, by considering their metabolic and functional traits, these phenotypes in disease states can be better distinguished.

**Figure 3:**
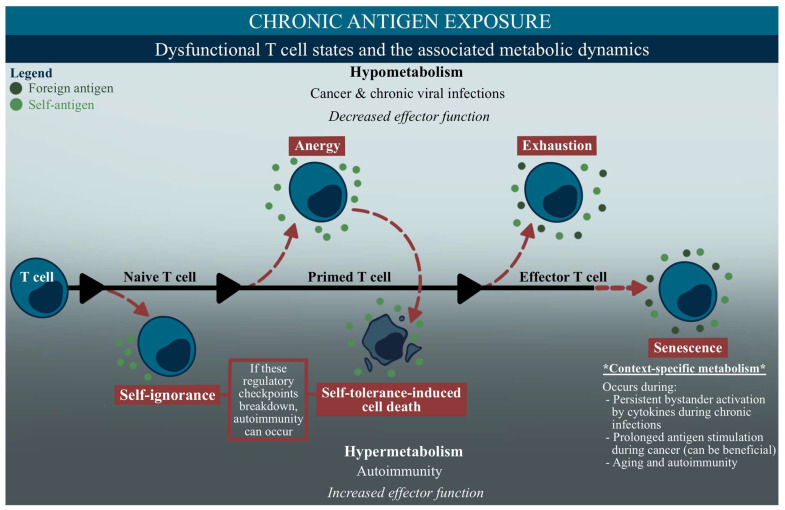
Integrated sequential diagram of the potential T cell dysfunction and correlated metabolic dynamics during chronic antigen exposure. This diagram depicts a T cell throughout the differentiation stages, and the altered phenotypes that can occur at each stage (in red), with a vertical gradient that illustrates the hypometabolic states at the top, and the hypermetabolic states at the bottom [97,114,115]. The legend at the top left denotes foreign antigen exposure (dark green circles), seen in cancer and chronic viral infections, and self-antigen exposure (light green circles), seen in autoimmunity and cancers [112,114,115]. The two phenotypes at the bottom, self-ignorance- and self-tolerance-induced cell death, are regulatory checkpoints that enforce T cell tolerance, and are typically described as dysfunctional if they do not occur within the proper context; the breakdown of these mechanisms can cause an autoimmune response [114,115,116,117]. Anergy, another tolerogenic response, is a hyporesponsive state that occurs following antigen exposure, and is driven by a deficient co-stimulation in the T cell receptors [112]. Anergic cells are in a hypometabolic state during chronic viral infections and cancer, exhibiting a decreased effector function and proliferation; however, within the context of self-antigens and autoimmunity, anergy can help prevent T cell pathogenicity before the effector stage begins, and the breakdown of anergic mechanisms can lead to an autoimmune response [55,112]. In effector cells, exhaustion and senescence can both limit cell responses, but through different mechanisms. While both states can be hypometabolic, with a low proliferative capacity, senescent cells are more widespread across the disease and metabolic capabilities, with the ability to maintain some level of effector function [112,118,119,120,121].

A common denominator of the dysfunctional T cell phenotypes discussed in this section is chronic antigen exposure. Persistent exposure to either self or foreign antigens is a trait in cancer, chronic viral infections, and autoimmunity, and the associated metabolic dynamics during these illnesses can vary, typically based on the effector function (Figure 3) [97]. Classically, the metabolic flexibility in effector T cells helps control the immune challenge presented, using multiple fuel sources in nutrient-limiting or -rich environments to help fight the infection [97]. However, during chronic activation, metabolic flexibility can be lost, and induce hypo- or hypermetabolic states. 

As depicted in the upper section of Figure 3, a hypometabolic state, or reduced metabolic activity, will display limited access to metabolic pathways that are essential for the effector function, ultimately leading to a weakened immune response in cases such as cancers and chronic viral infections [97]. Terms such as anergy and exhaustion are typically associated with this state and these diseases (Table 1, Figure 3) [112]. Meanwhile, hypermetabolic states, such as what is seen in autoimmunity, will increase a cell’s effector function, by utilizing large amounts of fuel for biosynthesis [97]. Deficits in the immunotolerance pathways, such as self-ignorance or anergy that leads to self-tolerance, can induce an autoimmune response (Table 1, Figure 3) [114,115,116]. Interestingly, cellular senescence, which continues to baffle scientists with its wide-ranging induction pathways and dynamic functions, has been observed in autoimmunity, cancer, and chronic viral infections, consequently displaying a variety of metabolic features that produce context-specific phenotypes in disease (Table 1, Figure 3) [118,119,120,121]. 

These states are summarized in Table 1, and are illustrated in a temporal fashion in Figure 3 [113,114,115,117]. The complexity of these immune cell phenotypes is further compounded by the overlap or progression of some diseases, such as when chronic viral infections lead to other conditions, including autoimmunity. This can occur with certain viruses, such as SARS-CoV-2 and EBV, which have developed mechanisms involving the molecular mimicry between viral antigen factors and human tissue antigens that can trigger autoimmune responses in individuals with a virally-induced chronically activated immune system [122,123,124]. The involvement of an autoimmune response in ME/CFS has been investigated in the past, but with conflicting findings. For instance, multiple studies have reported the presence of autoantibodies against nuclear structures in a subset of ME/CFS patients, with varying incidence rates, ranging from 13% to 68% [125,126,127,128,129]. Similarly, other studies have described the presence of various autoantibodies against neurotransmitters and markers of autoimmunity, albeit inconsistently in the literature, and only in subsets of ME/CFS patients [127,128,130]. Although the complex progression and intersection of these immune cell states pose significant challenges in studying diseases such as ME/CFS, this review will not delve further into discussing deficits in self-ignorance- or tolerance-induced cell death that could underscore the subset of patients potentially exhibiting autoimmune responses. Rather, the remaining section will concentrate on anergy, exhaustion, and senescence, examining their context within the metabolism and function, and their comparisons to ME/CFS.

## 3. Anergy

**Figure 4:**
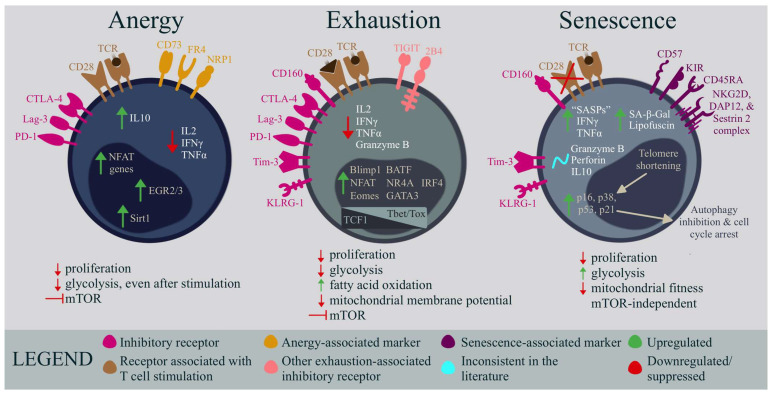
The surface markers, functional characteristics, transcription factors, and metabolic dynamics associated with T cell anergy, exhaustion, and senescence. While T cell anergy, exhaustion, and senescence have some overlapping features, these phenotypes differ in their transcriptional landscapes and mechanisms of action, and the metabolic switching that drives these states. These states can exist in both CD4+ and CD8+ T cells; however, anergy is more commonly associated with CD4+ T cells, while exhaustion is typically linked to CD8+ T cells, which is what is represented in this figure [136,137,138,140,141]. Of note, the middle panel of this figure illustrates the transcription factor dynamics in exhausted T cells, displaying the variable expression dependent on the stage of exhaustion; for example, TCF-1 plays a significant role during the progenitor stages of T cell exhaustion [142]. The senescent state portrayed here represents both the CD4+ and CD8+ T cells. However, research indicates that CD4+ T cells exhibit a greater resistance to aging than CD8+ T cells, due to their higher metabolic diversity, including an enhanced lipid and glucose uptake [143]. This flexibility allows CD4+ T cells a reduced senescence susceptibility, and slightly improved proliferative capabilities upon becoming senescent [143].

Anergy occurs when T cells do not receive the necessary signals for activation during stimulation, and fail to develop into functional effector cells [112]. This can occur when cells receive TCR stimulation, but no co-stimulation (by means of CD28), or through CTLA-4-associated T cell function-dampening (Figure 4, Table 1) [55,112]. In chronic viral infections, anergy is less common than other forms of dysfunction, such as exhaustion, but anergy could play a role when viruses use genetic strategies that directly inhibit the costimulatory pathways in antigen-presenting cells [55]. Anergy-induced non-responsiveness occurs swiftly during the initial antigen stimulation. Cells experiencing anergy exhibit a reduced effector function and anti-proliferative qualities, and the hallmark of this phenotype is their cell cycle arrest at the G_1_/S phase [131]. The identifying markers of anergy have yet to be clearly defined; however, some surface markers, such as CD73, FR4, Lag3, CTLA-4, PD-1, and NRP1 are associated with the CD4+ T cell anergic regulation and function (Figure 4) [114,131]. Additionally, anergy results in a reduced production of TCR-induced cytokines such as IL-2, IFNγ, and TNFα, and a transcriptional profile distinct from that of an exhausted phenotype, with upregulated NFAT, EGR2/3, and Sirt1 expression (Figure 4) [132,133,134,135]. While this state is classically defined for CD4+ T cells, CD8+ T cells seem to also become anergic and nonresponsive in a primed state, but with slightly different characteristics and mechanisms; for example, CD8+ T cells lacking CD4+ T cell assistance have a higher likelihood of becoming anergic, and anergic CD8+ T cells may not require CTLA-4, but actually necessitate CD28 co-stimulation to develop [136,137,138]. CD8+ anergic T cells also have an impaired proliferation and IL-2 production, but seem to retain the baseline production of IFNγ and TNFα [139].

In functional cells, CD28 signaling, facilitated through mTOR, upregulates metabolic machinery, such as amino acid and glucose transporters, transferrin receptors, and glycolysis [112]. During CD4+ T cell anergy, the mTOR and RAS/MAPK pathways are suppressed, which can lead to a decrease in these metabolic components (Figure 4) [132,144]. Deficits in glycolysis are also seen during CD8+ T cell anergy [139]. Additionally, CD4+ T cell starvation through nutrient blockade has also been shown to lead to anergy induction, consequently causing these cells to be metabolically deficient. This impairs the cell’s effector response, maintaining its hyporesponsive nature even upon future full activation (Figure 3 and Figure 4) [145]. 

### Comparative Analysis of Anergy and ME/CFS T Cells 

In ME/CFS T cells, basal glycolysis is reduced, the mitochondrial membrane potential is decreased, and extracellular fatty acids abound, resembling some metabolic features of anergy. However, while anergy can be rescued, in part, using IL-2 treatment that would stimulate the mTOR pathway, the metabolic differences remain after activating ME/CFS CD8+ T cells with IL-2 and a CD3/CD28 cocktail [91,139,146]. It may be that other metabolic-modulating cytokines, such as IL-7, IL-15, or IL-10 could be altering glycolysis and the subsequent effector functions in ME/CFS [147]. An increased level of IL-10, which suppresses cytokine production in both CD4+ and CD8+ T cells, has been reported in ME/CFS, and also in anergic cells (Figure 4) [16,148]. Interestingly, blocking IL-10 in a commonly studied chronic viral infection, LCMV, cleared the viral infection, and enhanced both the CD4+ and CD8+ T cell response [149,150]. However, ME/CFS plasma cytokine analysis has not definitely shown a decreased IL-2 production compared to healthy controls, as is seen in an anergic state; however, cytokine studies in ME/CFS are often inconsistent [93,151,152,153,154,155]. 

In contrast to anergic T cells, which cannot engage glycolysis even after full activation, our findings demonstrate that both CD4+ and CD8+ T cells from ME/CFS patients are capable of increasing glycolytic activity after activation (Figure 4) [91,145,156,157]. This suggests that chronic antigen stimulation in ME/CFS patients does not lead to the maintenance of an anergic state, where glycolysis cannot be engaged for T cell function. Additionally, while anergy would typically reduce the number of glucose transporters, we did not see a decrease in the GLUT1 in either the CD4+ or CD8+ ME/CFS T cell populations at rest; rather, the CD8+ T cells were able to upregulate GLUT1 at the same level in ME/CFS, compared to the control cells, after stimulatory factors were added [91]. It could be that intracellular mechanisms are responsible for the decreased glycolysis observed in ME/CFS, or that alternative glucose transporters are downregulated, independent of the GLUT1 abundance. Interestingly, however, one study investigating autophagy in ME/CFS serum samples demonstrated elevated levels of phosphorylated ATG13, which restricts autophagy in active cells [158]. If this translates to primed T cells in an anergic state (or effector T cells in another dysregulated state), the lack of autophagy could maintain cells with aberrant features (Figure 4). 

The characterization of surface markers and transcriptional features related to anergy is limited in ME/CFS. One study using flow cytometry found significantly increased CD4+CD73+CD39- and Treg populations in ME/CFS whole-blood samples, while a separate ME/CFS Treg study found a trend of higher CD39+CD73+ frequency in this illness, compared to healthy controls, further supporting the theory of anergic features in ME/CFS CD4+ T cells [94,159]. A gene expression study reported an elevated expression of four NFAT-related genes, while another analysis of transcription-factor binding sites in PBMCs following microarray analysis and RT-PCR showed an upregulation of EGR2/3; however, this latter finding was only associated with EBV-linked ME/CFS infection or reactivation [160,161,162]. Contrary to the anergic transcriptional features, increases in microRNAs that regulate Sirt1 indicate that the Sirt1 expression may be lower in ME/CFS PBMCs [163]. Further research is required, specifically examining the single-cell transcriptional state of the T cells in ME/CFS, to ascertain the presence of anergic cells within any T cell subsets, as opposed to other dysfunctional phenotypes, and their potential contribution to immune cell impairment in ME/CFS.

## 4. Exhaustion

T cell exhaustion is a state of dysfunction that occurs during persistent antigen stimulation in chronic viral infections or cancer [164]. Exhausted CD8+ T cells are characterized by a progressive loss of effector functions, decreased proliferation, and a sustained expression of inhibitory receptors, such as PD-1, CTLA-4, Lag-3, TIGIT, KLRG-1, 2B4, CD160, and Tim-3 (Figure 4) [164,165,166]. Unlike anergy, exhaustion occurs after the primary co-stimulation has occurred and the T cells have differentiated into effector cells. Functional capabilities, such as IL-2, IFNγ, TNFα, and Granzyme B production are gradually lost, and memory T cells do not develop properly (Figure 4) [114,131]. Moreover, IL-10 signaling regulates chronic infection progression and, while it is unclear whether or not exhausted T cells directly produce IL-10, increased plasma IL-10 levels do promote and maintain T cell exhaustion [167]. Epigenetic and transcriptional changes during chronic antigen stimulation drive the limited functional state, and transcription factors such as NFAT, BLIMP1, EOMES, BATF, NR4A, GATA3, and IRF4 are all upregulated (Figure 4) [114,168]. Additionally, different stages of exhaustion, from early progenitor to irreversible terminally differentiated exhausted CD8+ T cells can be teased apart by determining the levels of TCF1, T-bet, and Tox transcription factors in CD69+/- cell populations (Figure 4) [142,168]. Evolutionarily, the immune system is thought to employ T cell exhaustion as a mechanism to balance the immune response in the face of persistently replicating viruses, thereby preventing autoimmune reactions; however, this process can result in reduced immunity, and the inadequate clearance of chronic viral infections in the host, ultimately leading to detrimental effects on the body [169]. 

Metabolism in CD8+ T cell exhaustion has been studied by multiple groups, and it has been suggested that an abnormal metabolic regulation not only characterizes this phenomenon, but also actively drives this immune cell state [157]. Metabolically, mTOR is suppressed in T cell exhaustion and, as a result, these cells exhibit a reduced glucose uptake and glycolysis, as well as increased fatty acid oxidation (Figure 4) [74]. Blocking CPT1a, a fatty acid transporter, reduced mitochondrial respiration in early exhausted T cells, indicating that, while fatty acid usage may be a survival mechanism in glucose-deprived conditions, it still may not be enough to sustain a strong immune response [170]. Additionally, the spare respiratory capacity, mitochondrial mass, and mitochondrial membrane potential are all reduced in exhausted CD8+ T cells (Figure 4) [112]. The loss of mitochondrial function leads to the accumulation of toxic metabolites and reactive oxygen species (ROS), which can further exacerbate T cell exhaustion [171]. 

The prevalence of CD4+ T cell exhaustion remains a debated topic, as many studies have focused predominantly on CD8+ T cell exhaustion. However, not only can CD4+ T cells exhibit an exhausted phenotype, defined by the expression, or lack, of the associated inhibitory receptors and cytokine production levels, but classic CD4+ T cells also play a pivotal role in preventing CD8+ T cell exhaustion, by promoting the CD8+ effector function [140,141]. Unfortunately, there is limited research exploring the proliferation, metabolic dynamics, and transcriptional/epigenetic changes in exhausted CD4+ T cell populations, further emphasizing the need for additional investigations in this area.

### Comparative Analysis of Exhaustion and ME/CFS T Cells

In ME/CFS, the metabolic profile of CD4+ and CD8+ T cells that we observed is consistent with T cell exhaustion. In particular, glycolysis is inhibited, and the FAO is promoted in both the cell populations in ME/CFS, and exhausted cells [74,91,92]. The mitochondrial membrane potential was also reduced in the ME/CFS CD8+ T cells, similar to an exhausted phenotype [91]. The identification of the increased markers and damage associated with oxidative stress in individuals with ME/CFS, particularly following exercise or during relapse, provides additional support for the hypothesis of reactive oxygen species (ROS) accumulation and the subsequent aggravation of an exhausted T cell phenotype, in this condition [84,172,173,174]. 

Flow cytometric analyses in ME/CFS have shown increased CD4+ PD-1+ cells, an increase in the Th17 population, and a decrease in the cytokine production in other T cell populations, all features of exhausted immune cell states [93,96,164,175]. Moreover, decreased proliferative capabilities in ME/CFS T cells, and increased ME/CFS plasma IL-10 have also been observed [16,93]. Further analysis of the related surface markers is needed to better identify this phenotype in ME/CFS; however, decreased CD8+KLRG1+ and CD4+KLRG1+ population frequencies have been noted in ME/CFS subjects [176]. It bears mentioning that the variety of inhibitory receptors, and their diverse combinations that can result in T cell exhaustion make these ME/CFS surface marker studies difficult to interpret. 

In addition to the aforementioned ME/CFS gene-expression study with elevated NFAT-related genes, a DNA methylation study in ME/CFS PBMCs revealed a hypo-methylated state in the IRF4 enhancer region, suggesting an upregulation of this exhaustion-related protein [160,177]. Otherwise, the transcriptional landscape of ME/CFS T cells, as it relates to an exhausted state (illustrated in Figure 4), has yet to be fully assessed, but would be valuable in determining the state of these cells.

## 5. Senescence

Senescence, or cellular aging, is a universal state seen in many cell populations during stress and age that, in the immune system, can exhibit paradoxical effects on adaptive immunity (Figure 3 and Figure 4) [178]. This state is characterized by telomere shortening, the loss of CD28 and CD27 expression, CD45RA re-expression, and cell cycle arrest in both CD4+ and CD8+ T cells (Figure 4) [118]. Although aging is primarily associated with the onset of senescence, this phenomenon is also regarded as a favorable mechanism for preventing the growth of tumors in cancer [131]. Furthermore, senescent T cells have also been observed in individuals who are younger and suffer from autoimmune disorders, or persistent viral infections, indicating that its onset may be triggered by the persistent stimulation of the associated cells [131]. Indeed, short telomeres have been observed in highly differentiated CD4+ and CD8+ T cells in individuals with varying chronic viral infections, such as EBV, HIV, HBV, and HCV [169,179,180,181]. As opposed to immune cell exhaustion, senescence seems to be an omnipresent outcome of extensive replication, rather than a consequence of antigen-specific activation or stimulatory/inhibitory conditions; however, an elevated Tim-3 abundance can also be found on the cell surface of senescent T cells (Figure 4) [182,183,184]. 

Despite the loss of proliferative capacity, seen to greater extents in senescent CD8+ T cells, senescent CD4+ and CD8+ T cells maintain the ability to generate senescence-associated secretory phenotype (SASP) factors, rich in pro-inflammatory molecules such as IFNγ and TNFα (Figure 4) [143,185,186,187,188,189,190]. On the other hand, this state is associated with inconsistent levels of IL-10, perforin, and Granzyme B, typically dependent on the disease or the stage of senescence (Figure 4) [191,192]. Other defining features of T cell senescence include the acquisition of innate, NK cell-like markers, such as KLRG-1, KIR, and CD57, along with the procurement of an NKG2D, DAP12, and Sestrin 2 complex that plays a large role in inducing cytotoxicity in senescent cells (Figure 4) [118,193,194,195,196,197]. Senescence-associated β-galactosidase (SA-β-Gal) and lipofuscin accumulation is also a hallmark of this phenotype (Figure 4) [186,198,199]. The transcriptional changes in this state lead to an increased translation of cell cycle arrest proteins, such as p16, p21, and p53 (Figure 4) [131,200]. In addition, the p38 upregulation in senescence inhibits autophagy in the CD4+ and CD8+ T cells (Figure 4) [190,201,202]. 

The metabolic dynamics in T cell senescence are quite different from the other dysfunctional states discussed in this review. For example, stimulated senescent CD8^+^ T cells exhibit impaired mitochondrial fitness and biogenesis and, consequently, tend to rely on glycolysis over OXPHOS for energy (Figure 4) [190]. CD4+ senescent T cells similarly rely on glycolysis and lipids for their maintenance [143]. The failure of autophagy in senescent cells results in the accumulation of damaged organelles, such as the dysfunctional mitochondria mentioned above. Consequently, these damaged mitochondria generate reactive oxygen species (ROS), leading to cellular impairments, such as DNA damage. Classically, the T cells that can perform mitochondrial biogenesis exhibit a higher tolerance to metabolic stress, known as the spare respiratory capacity [203]. Therefore, it follows that senescent CD8+ T cells possess a considerably diminished spare respiratory capacity compared to healthy memory subsets, rendering them metabolically unstable [190]. Meanwhile, lipid metabolism has not been thoroughly investigated in all instances of senescence; however, in the context of cancer, it appears to be unbalanced. One study that investigated CD4+ and CD8+ senescent T cells in tumors observed altered cholesterol synthesis, fatty acid oxidation, and fatty acid synthesis enzyme expression, which increased some lipid species concentrations, while reducing others [204]. Although mTOR inhibition has shown promising results in old mice, it does not impact the effector functions of human senescent CD8+ T cells. Specifically, rapamycin, a commonly used mTOR inhibitor, reduces IFNγ production in less-differentiated T cells, but this effect is not observed in senescent CD8+ T cells [205,206]. These findings suggest that other mechanisms, beyond the mTOR pathway, are likely involved in the regulation of the metabolic dynamics of senescence. 

### Comparative Analysis of Senescence and ME/CFS T Cells

Although senescent T cells usually display a rise in glycolysis, and a decrease in the spare respiratory capacity, such findings are not observed in ME/CFS [91]. However, it is possible that our research might have been influenced by the fact that we investigated the total CD4+ or CD8+ T cells, instead of specifically studying the terminally differentiated memory-like T cells that exhibit senescence. Meanwhile, the fatty acid oxidation was increased in the ME/CFS effector and memory CD4+ and memory CD8+ T cells, which could be consistent with senescence. ME/CFS cytokine observations also appear to agree with the senescence phenotypes, as SASP-like pro-inflammatory cytokines, such as TNFα, TGFβ, IL-1α, IL-1β, IL-4, IL-6, and IFNγ positively correlate with the ME/CFS disease severity; however, the overall analysis of cytokines in ME/CFS shows varying degrees of consistency [93,151,152,153,154,155]. Moreover, the autophagy inhibition findings and oxidative stress/damage in ME/CFS also appear to agree with similar characteristics in T cell senescence (Figure 3 and Figure 4) [84,158,172,173,174,190]. 

The surface markers still need further investigation, but it is worth noting that in two studies, the frequency of CD4+CD28- and CD8+CD28- populations, like that of senescent cells, were not increased in a cohort of ME/CFS PBMC samples, compared to healthy controls [93,207]. Rather, a third study found decreased proportions of terminally differentiated effector CD8+ T cells in ME/CFS, compared to a healthy cohort [208]. Another flow-cytometric analysis found a significantly lower abundance of CD57, a common senescence marker, in ME/CFS CD3+ T cells, and a slightly lower frequency of CD3+ CD57+ cells, compared to the healthy control samples [209]. Conversely, in moderately severe ME/CFS patients from a separate study, the ME/CFS T cells showed an increased KIR2DL5 expression (part of the KIR family) on CD4+ cells, but decreased CD8+KLRG1+ and CD4+KLRG1+ population frequencies [176].

A report from 2018 on ME/CFS DNA from whole blood revealed significantly different telomere lengths compared to healthy controls, suggesting some cells in this heterogenous cell population have shortened telomeres not associated with general aging (female participants <45 years old were a large contributor to the phenotype) [210]. A separate DNA methylation gene ontology analysis in total PBMCs indicated that the P38 MAPK signaling pathway was upregulated in ME/CFS, compared to controls [211]. In contrast, an elevated production of NF-κB has been detected in ME/CFS lymphocytes, which antagonizes p53 transcription, the opposite of what is seen in T cell senescence [212,213]. The heterogeneity of these ME/CFS studies’ cell populations makes it difficult to confirm this state in one specific immune cell population, highlighting the need for single-cell transcriptomic analyses in this disease.

## 6. Comparative Analysis of Dysfunctional NK Cell States and ME/CFS NK Cells

It remains unclear whether exhaustion, anergy, and senescence in NK cells represent unique types of dysfunctional states. Generally, NK cell dysfunction, typically marked by a decline in the effector function or proliferation, is assessed by the IFNγ and Granzyme B production, but these markers are very generic, and cannot distinguish between the three phenotypes listed above [182]. Judge et al. created an elegant comprehensive review of the current state of investigations into exhaustion, anergy, and senescence in NK cells [182]. 

Anergy has not been studied extensively in NK cells, but has been hypothesized in depth in the aforementioned review. For example, NK cells, which require a three-signal sequence for maximal activation and proliferation, may become anergic following an insufficient activating signal, or a strong stimulus without an adequate co-stimulation [214]. Proposing a comparable process to that of anergic T cells, NK cells would become anergic if CD137 signaling were not engaged at the time of the NK–antigen-presenting-cell (APC) contact [182,214]. Of note, a tumor study defined NK cell anergy precisely in this manner, whereby anergic NK cells were accompanied by an impaired early signal transduction [215]. The proliferative capabilities during this understudied condition in NK cells are unknown, but the cytotoxicity would very likely be reduced as a consequence of this hypometabolic, hyporesponsive state [182].

NK cell split anergy, or the loss of one function with the corresponding gain of another effector function, has been observed following the ligation of CD16 [216,217]. This anergy-adjacent cell state is characterized by the loss of CD16 expression, with the addition of cytokine secretion abilities, leading to the increased control of cancer stem cells [216]. While pro-inflammatory cytokines can boost the NK cell effector function and proliferation, there are still signs of anergy in these environments, as NK cells work to establish a balance between recognizing targets effectively, and maintaining tolerance by developing additional immunoregulatory mechanisms [218,219]. 

NK cell exhaustion has drawn much more attention for its potential to enhance anti-tumor properties through NK-specific immunomodulatory therapies, making it a promising area for further investigation in other diseases, as well. NK cell exhaustion is identified through a decrease in the effector function, and an increase in exhaustion-associated markers such as PD-1, TIGIT, TIM-3, or LAG-3 [220,221,222,223,224,225]. The reduced IFNγ expression by NK cells is the most notable effector function in identifying exhausted NK cells, while the increased expression of a conventional exhaustion-associated marker confirms this state. There is, however, controversy about PD-1 being an exhaustion marker on NK cells, and studies have demonstrated that TIGIT, TIM-3, and LAG-3 may be more critical markers [182,225,226]. Evaluating the effectiveness of anti-PD-1 therapy on PD-1+ and PD-1- subsets is essential to confirm the specificity of these findings. Flow-cytometric gating to determine a positive exhausted NK population poses a challenge, and can result in a widely variable expression of exhaustion markers, as evidenced by the significant variability in PD-1 expression on intra-tumoral NK cells in mice [182,227,228]. Checkpoint blockade therapy, targeting the PD-1/PD-L1 pathway, shows significant anti-tumor effects, although there is no direct confirmation as to whether this PD-1+ NK cell population represents an exhausted subset, or an anergic state [182,229]. 

Although the precise mechanisms underlying NK cell exhaustion in tumors and chronic infections are not well defined, recent research suggests that multiple negative regulatory pathways may contribute to this exhausted state in NK cells [230,231]. These pathways include the aberrant signaling of NK cell receptors, and suppression by regulatory cells or soluble factors in the microenvironment [230]. For example, continuous IL-15 exposure has been shown to exhaust NK cells by reducing the mitochondrial respiration, exhibiting decreased fatty acid oxidation [231]. Meanwhile, the transcriptional landscape in exhausted NK cells differs from T cell exhaustion features, as a repressed expression of Eomes and T-bet was found to partially account for an exhausted NK cell phenotype [232].

While the characteristics of NK cell senescence have not been fully exposed, it is assumed that senescent NK cells are also characterized by an increase in pro-inflammatory SASP factor production, and a decreased cell proliferation. In one study of the implications of aging on NK-cell senescence, researchers theorized that senescence could lead to an increased viral infection susceptibility, diminished antimicrobial functions, and an increased unchecked malignant cell persistence [233]. The lifespan of NK cells, which would help to determine the senescent features in this cell population, is not well-defined. While NK cells are less susceptible to the effects of aging compared to other lymphocytes, Gounder et al. showed that they still undergo age-related dysfunction, as younger donors’ NK cells expanded more than those from older donors [234]. Similarly, two other studies found age-related differences in NK cell murine experiments, with a decrease in the total and mature NK cells in the peripheral tissues, and an accumulation of mature NK cells in the bone marrow, of aged mice compared to younger mice [235,236]. 

In a study characterizing aging NK cells in vitro, investigations revealed an increased inhibitory receptor (NKG2A) expression, and decreased maturation marker (CD57) levels during an 8-week culture, distinct from T cell senescence [237]. Additionally, no variance was observed in the IFNγ generation, or cytotoxicity toward target cells, in the CD57 positive and negative subgroups [237]. Overall, the findings of these studies should be considered in the context of cancer or generalized aging, and further research is needed to understand chronic viral infection-associated NK cell senescence. Although our understanding of NK cell senescence with respect to metabolism and transcription is currently limited, we can infer that they are likely dynamic and context-specific profiles, such as what is seen in T cell senescence.

An understanding of the dysfunctional state of the NK cells in ME/CFS remains elusive, due to the absence of distinct markers for identifying these dysfunctional phenotypes. Despite the observed decreased NK cell cytotoxicity, it is uncertain if this is due to anergy, exhaustion, or senescence. The increased fatty acid oxidation levels in the ME/CFS NK cells seen in our study suggest that exhaustion may not be the dysfunctional state in this disease, but identifying just one metabolic feature is not enough to characterize the phenotype of these cells [92]. Lower frequencies of NKG2C+ (an activating receptor) NK cell populations have been observed in ME/CFS NK cell flow-cytometric analyses, which could contribute to their reduced function; however, the same study reported no difference in the senescence-related NKG2A+ and CD57+ NK cell subsets in ME/CFS, compared to the healthy control cell populations [238]. There is still a lack of knowledge on anergy or senescent NK cell metabolism, transcription, and function, to properly identify which dysfunctional phenotype may be present. Thus, additional research is needed to fully comprehend the mechanisms responsible for NK cell dysfunction in ME/CFS.

## 7. Conclusions

ME/CFS is a serious chronic illness characterized by significant disability, an ambiguous pathology, and a suspected initiation by persistent viral stimulation. CD8+ T cells, CD4+ T cells, and NK cells are crucial to the immune system, with metabolic features that are essential for an effective response to viral challenges. While the exact metabolic shifts vary, depending on the cell type and context, there is evidence to suggest that metabolic dysfunction is, in part, driving the dysfunctional immune cell states in ME/CFS. The precise characteristics of these dysfunctional states in ME/CFS are uncertain, and it is plausible that several distinct dysfunctional states are operating in various immune cell populations in ME/CFS, affecting each other, and intensifying the altered phenotypes. For example, during chronic infections, diverse NK cell functions can trigger T cell exhaustion [239]. 

The heterogeneity in the presentation of ME/CFS, and the conflicting results reported in the literature may be attributed to variations in the immune cell states at different stages of the disease progression; a person with ME/CFS for seven years may exhibit distinct immune cell dysfunction and characteristics compared to someone who has been suffering from the condition for over 20 years. Not only that, but the immune system of individuals with ME/CFS can undergo frequent changes over a single year. A longitudinal study examining cytokine production in ME/CFS patients tested at 0, 6, and 12 months for NK cell cytotoxicity and cytokine secretion showed inconsistent, and sometimes opposing, levels of TNFα, IFNγ, IL-10, IL-17A, and IL-2 across the three timepoints in the same individual [155]. 

This review comprehensively assesses the existing ME/CFS T and NK cell literature, and suggests a potential link to an exhausted CD8+ T cell state; however, the specific status of CD4+ T cells and NK cells remains enigmatic. Nevertheless, the resemblance of ME/CFS to other chronic viral infections, evident in the hypometabolic immune cell state and reduced effector functions, suggests the existence of dysfunctional states within the T and NK cell populations. Identifying the dysfunctional states, including anergy, exhaustion, and senescence, within individual immune cell populations of ME/CFS can provide valuable insights into potential therapeutic targets, as some of these states can be reversible. Further, accurately characterizing these states, once identified, will be crucial to tailoring treatments appropriately. For instance, in exhausted cells, diverse inhibitory receptor co-expression can occur during chronic infections, highlighting the need to precisely define T cell properties. to enhance beneficial functions without exacerbating the immunopathology [55]. Further research is necessary to comprehend the immunometabolic changes and functional consequences observed in ME/CFS, particularly considering the overlapping features of dysfunctional states, and the gaps in the existing ME/CFS research. This understanding is essential to devising innovative therapeutic interventions to improve patient outcomes in ME/CFS.

## Figures and Tables

**Table 1:** Characterization of terminology associated with dysfunctional T cells during chronic antigen exposure.

Term	Human Diseases Associated with this State	Short Description
(Loss of) self-ignorance	Autoimmunity	Antigen-inexperienced autoreactive T cells that persist in the periphery due to low antigen expression or physical isolation. If ignorance is overcome, this can induce autoimmunity.
(Loss of) self-tolerance	Autoimmunity	T cells that express high-affinity receptors to self-antigens, and will induce cell death once activated. If the body cannot regulate these cells, the breakdown of this state can cause autoimmunity.
Anergy (and the loss of this mechanism)	Autoimmunity Chronic viral infections Cancer	T cells that are unable to mount an immune response, driven by sub-optimal co-stimulation (CD28) and/or an increased inhibitory receptor abundance (CTLA4) during a primed cell state. While an anergic T cell can be found in chronic viral infections and cancer, a breakdown of this tolerance mechanism can induce autoimmunity.
Exhaustion	Chronic viral infections Cancer	An unresponsive T cell state characterized by a progressive loss of effector functions, and high levels of inhibitory factors during prolonged antigen exposure.
Senescence	Autoimmunity Chronic viral infections Cancer	An irreversible T cell state that occurs during aging, chronic infection, and cancer, differing from exhaustion by having low levels of inhibitory receptors. Proliferation is lost, apoptosis is inhibited, and the cell releases a mixture of molecules that can contribute to inflammatory pathways.

## Data Availability

Not applicable.
